# The role of exercise-induced myokines in promoting angiogenesis

**DOI:** 10.3389/fphys.2022.981577

**Published:** 2022-08-26

**Authors:** Chao Qi, Xianjing Song, He Wang, Youyou Yan, Bin Liu

**Affiliations:** Department of Cardiology, Second Hospital of Jilin University, Changchun, China

**Keywords:** myokine, exercise, angiogenesis, ischemic diseases, cytokines

## Abstract

Ischemic diseases are a major cause of mortality or disability in the clinic. Surgical or medical treatment often has poor effect on patients with tissue and organ ischemia caused by diffuse stenoses. Promoting angiogenesis is undoubtedly an effective method to improve perfusion in ischemic tissues and organs. Although many animal or clinical studies tried to use stem cell transplantation, gene therapy, or cytokines to promote angiogenesis, these methods could not be widely applied in the clinic due to their inconsistent experimental results. However, exercise rehabilitation has been written into many authoritative guidelines in the treatment of ischemic diseases. The function of exercise in promoting angiogenesis relies on the regulation of blood glucose and lipids, as well as cytokines that secreted by skeletal muscle, which are termed as myokines, during exercise. Myokines, such as interleukin-6 (IL-6), chemokine ligand (CXCL) family proteins, irisin, follistatin-like protein 1 (FSTL1), and insulin-like growth factor-1 (IGF-1), have been found to be closely related to the expression and function of angiogenesis-related factors and angiogenesis in both animal and clinical experiments, suggesting that myokines may become a new molecular target to promote angiogenesis and treat ischemic diseases. The aim of this review is to show current research progress regarding the mechanism how exercise and exercise-induced myokines promote angiogenesis. In addition, the limitation and prospect of researches on the roles of exercise-induced myokines in angiogenesis are also discussed. We hope this review could provide theoretical basis for the future mechanism studies and the development of new strategies for treating ischemic diseases.

## 1 Introduction

**FIGURE 1:**
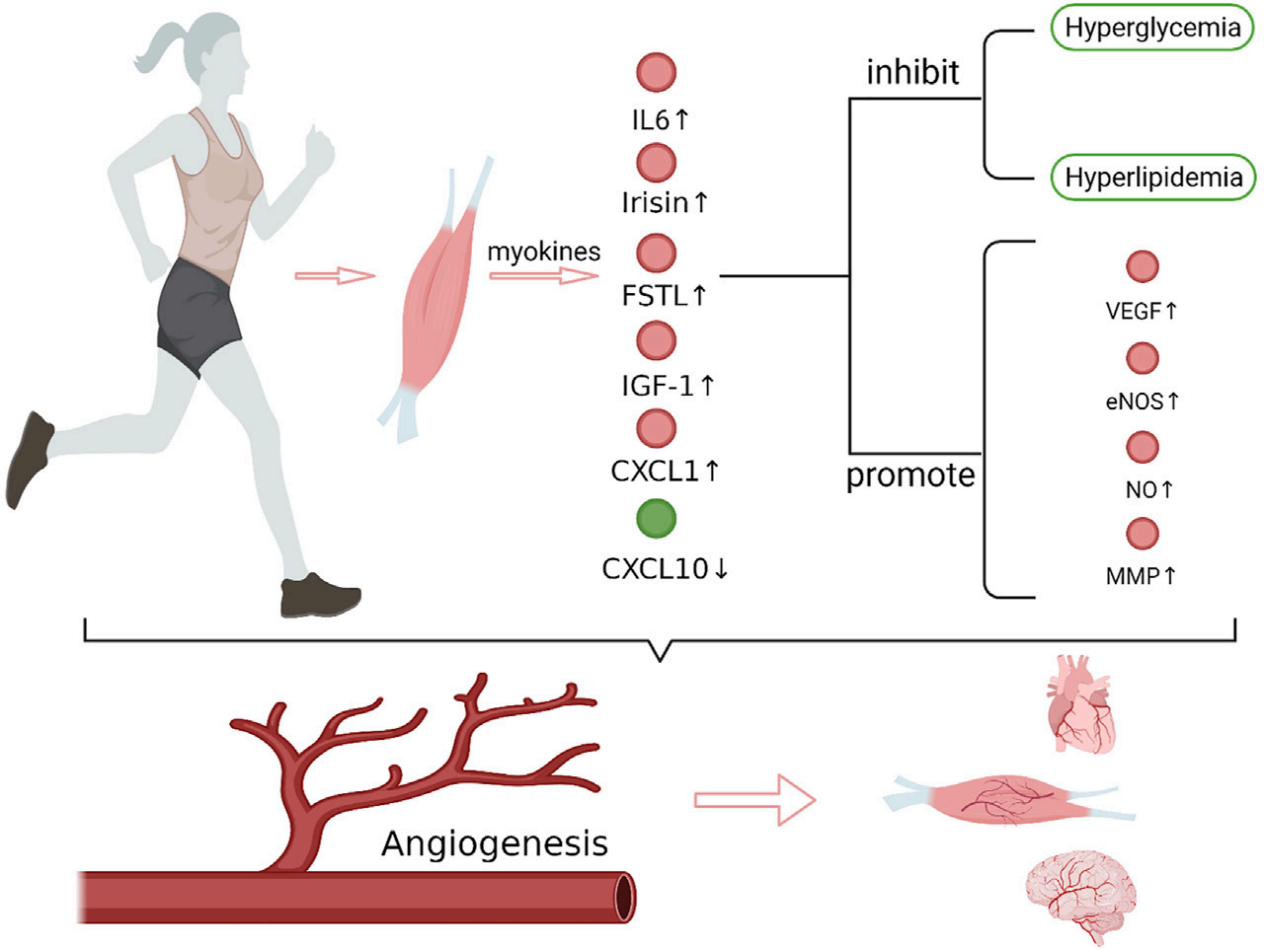
Exercise and exercise-induced myokines in promoting angiogenesis. After exercise, the expression of IL-6, irisin, FSTL1, CXCL1, IGF-1 that promote angiogenesis increased in muscles, while the expression of CXCL10, which inhibits angiogenesis, decreased. These myokines regulate the expression of angiogenesis-related factors, such as VEGF, eNOS/NO and MMP, which play important roles in endothelial cell matrix degradation, endothelial cell migration and lumen formation, and synergistically promote angiogenesis. In addition, myokines also improve hyperglycemia and hyperlipidemia. Together, these effects of myokines promote angiogenesis in brain, heart and muscle tissue. VEGF, vascular endothelial growth factor; eNOS, endothelial nitric oxide synthase; MMP, matrix metalloproteinase (Create with BioRender.com).

Ischemic diseases, including ischemic heart disease, ischemic stroke, and peripheral artery disease, are characterized by insufficient perfusion of tissues and organs caused by vascular stenosis or even occlusion. Inflammatory reactions, vasospasms, thromboembolisms, fat embolisms, and uneven blood distribution can all result in ischemic diseases, while atherosclerosis is the most common cause. Hypertension, hyperglycemia, and hyperlipidemia are important factors that promote atherosclerosis. When severe diffuse atherosclerosis occurs in cerebral vessels, coronary arteries, or peripheral arteries, surgical or medical treatment usually shows poor effect and the patients would have a poor prognosis. Exercise rehabilitation therapy can significantly improve the symptoms and prognosis of patients with ischemic stroke, ischemic heart disease, and peripheral artery disease (Treat-Jacobson et al., 2019; MacKay-Lyons et al., 2020; Pelliccia et al., 2021), and has already been used as a treating method along with medication and surgical operation. Skeletal muscle is the main organ involved in exercise, in the meantime, it is also the largest glycogen reserve organ and an important endocrine organ. Skeletal muscle can produce myokines that cause a series of biological effects through autocrine, paracrine, or remote secretion during exercise (Giudice and Taylor, 2017). It is reported that exercise can promote angiogenesis in the skeletal muscle, myocardium, and brain (Morland et al., 2017; Longchamp et al., 2018; Tryfonos et al., 2021). Neovascularization is an important mechanism for exercise to improve the clinical symptoms and prognosis of ischemic diseases. Some studies have shown that this effect of exercise is closely related to myokines. This review focuses on the current research progress of the mechanism underlying exercise-mediated promotion of angiogenesis through myokines (Figure 1).

## 2 Angiogenesis and current strategies for promoting angiogenesis

### 2.1 Angiogenesis related mechanisms: physiological and pathophysiological

In a narrow sense, angiogenesis refers to the process of degradation of the original capillary or microvascular endothelial matrix, the proliferation, migration and differentiation of endothelial cells, formation of a new vascular lumen in the form of budding, synthesis of new extravascular matrix, and the formation of new blood vessels (Diaz-Flores et al., 1994). While in a broader sense, angiogenesis includes the process of expanding, maturing, or forming arterioles containing a muscle layer on the basis of the original vascular lumen. At present, many cytokines regulating angiogenesis have been found, including vascular endothelial growth factor (VEGF), fibroblast growth factor (FGF), angiopoietin 1,2, transforming growth factor-β (TGF-β), erythropoietin, epidermal growth factor (EGF), interleukin-6 (IL-6), matrix metalloproteinase (MMP), and endothelial nitric oxide synthase (eNOS) (Table 1). Among them, VEGF is the most critical cytokine regulating angiogenesis. It participates in many processes of angiogenesis, such as proliferation and migration of endothelial cells, change of vascular permeability, and degeneration of extracellular matrix. MMP also plays an extremely important role in the degradation of collagen around microvessels in the early stage of angiogenesis. The complex formed by cell membrane-MMP and urokinase in cell pseudopodia can adhere to migrating endothelial cells and improve the migration rate and survival rate of endothelial cells (van Hinsbergh et al., 2006). FGF can promote endothelial cell migration, smooth muscle cell proliferation, and the gradual maturation of newly formed vascular lumen. Endothelial cells can synthesize NO under the regulation of eNOS. Under physiological conditions, NO can increase the level of cGMP in cells and affect the mRNA transcription process, and the angiogenic effects of FGF-2 and VEGF are closely related to the NO signaling pathway (Ziche and Morbidelli, 2000; Kivela et al., 2019).

**TABLE 1:** Function of angiogenesis-related cytokines in promoting angiogenesis.

Cytokines	Function
MMP	Degradation of collagen around microvessels in the early stage of angiogenesis
VEGF	Proliferation and migration of endothelial cells, change of vascular permeability, and extracellular matrix degeneration
FGF	Endothelial cell migration, smooth muscle cell proliferation, and the gradual maturation of newly formed vascular lumen
eNOS	Regulation of NO synthesis. The angiogenic effects of FGF-2 and VEGF are closely related to the NO signaling pathway

### 2.2 Current strategies for promoting angiogenesis

Angiogenesis is an effective method to improve blood perfusion in ischemic tissues and organs, and alleviate ischemic symptoms. Current strategies to promote angiogenesis for treating ischemic diseases (including those under development) include cell therapy, gene therapy, cytokine therapy, and exercise rehabilitation (Losordo and Dimmeler, 2004a, b). Strauer et al. (Strauer et al., 2002) transplanted autologous bone marrow monocytes into the inner wall of blood vessels of myocardial infarction patient through an interventional balloon, and found that these bone marrow monocytes cells can significantly improve perfusion in the ischemic area by promoting angiogenesis. Besides, transplantation of autologous bone marrow stem cells at the ischemic site in patients with peripheral arterial disease can also improve the symptoms caused by ischemia (Tateishi-Yuyama et al., 2002). At present, the targets of gene and cytokine therapy to promote angiogenesis mainly include VEGF and its receptors, FGF family proteins, and HIF-1. Among them, VEGF and FGF have been found to promote myocardial perfusion in phase Ⅰ or phase Ⅱ clinical trials in the treatment of patients with ischemic heart disease (Losordo and Dimmeler, 2004a). Although many animal experiments using gene therapy successfully promoted peripheral angiogenesis, phase Ⅱ or phase Ⅲ randomized controlled clinical trials of gene therapy for peripheral arterial diseases have shown both positive and negative results. Therefore, the application prospect of gene therapy to promote peripheral angiogenesis is unclear yet (Simon et al., 2022). Compared with these above strategies, exercise rehabilitation has been incorporated into many authoritative guidelines as an improvement method for many ischemic diseases (Treat-Jacobson et al., 2019; MacKay-Lyons et al., 2020; Pelliccia et al., 2021). Exercise-mediated improvement of ischemic symptoms is closely related to angiogenesis promotion. The EXCITE clinical trial showed that exercise rehabilitation can promote coronary collateral circulation growth in patients with stable angina pectoris (Mobius-Winkler et al., 2016). The capillary density per unit of skeletal muscle in young men with good exercise practice was significantly higher than that of untrained men (Hermansen and Wachtlova, 1971). After aerobic training, it was found that the levels of VEGF and capillary density in the mouse brain increased significantly (Morland et al., 2017). Therefore, exercise therapy plays an important role in improving the symptoms and prognosis of ischemic diseases, an in-depth discussion of the function and mechanism of exercise in promoting angiogenesis is of great significance for improving the therapeutic effect of exercise therapy, formulating individualized treatment plans, and also discovering new therapeutic targets.

## 3 Exercise-induced internal environment changes in the context of angiogenesis

### 3.1 The effect of exercise on glucose and lipid metabolism

Many studies have reported that the role of exercise in promoting angiogenesis is related to the changes of the internal environment after exercise. For example, hyperlipidemia and hyperglycemia can damage endothelial function, cause vascular endothelial inflammatory reactions, promote atherosclerosis formation, and inhibit angiogenesis. Exercise can significantly increase the metabolic rate of blood lipids and glucose. Clinical observation shows that aerobic training improves the blood lipid levels of patients with hyperlipidemia (Zhao et al., 2021). Obesity induced by a high-fat diet also has a negative effect on angiogenesis. Aerobic exercise can improve obesity and promote the expressions of myokine and VEGF (Shin et al., 2015). Besides, skeletal muscle accounts for the main part of glucose utilization under insulin stimulation, thus plays an important role in blood glucose control and metabolic homeostasis. During muscle contraction, the circulation of glucose transporter four in the cell membrane of skeletal muscle cells is increased, which enhances glucose uptake and reduces blood glucose concentration (Nedachi et al., 2008). In addition, exercise can improve insulin resistance in obese patients and improve glucose utilization in muscle tissue (Coffey and Hawley, 2007).

### 3.2 The effect of exercise on angiogenesis-related cytokines

Except for blood lipid and glucose metabolism, the expression level and distribution of angiogenesis-related cytokines are also regulated by exercise to promote angiogenesis (Bloor, 2005). Gavin et al. (Gavin et al., 2004) showed that the protein level of VEGF in muscle tissue was decreased after exercise, while the protein level of VEGF in the serum and myocardium was increased (Yazdani et al., 2020; Dastah et al., 2021). The difference of VEGF protein level in different tissues after exercise may be due to the increased blood volume in muscle during exercise, which resulted in VEGF protein redistribution. In addition, NO synthesis is also changed significantly during exercise (Dyakova et al., 2015). NO was first considered as an inflammatory factor that regulates vasoconstriction. Later, it was found that NO plays an important role in angiogenesis. NO synthesis is regulated by eNOS, and the effect of NO/eNOS is closely related to VEGF. The protein level of eNOS in the myocardium was increased after exercise, which was correlated with increased expression of VEGF (Iemitsu et al., 2006). The mRNA expression of VEGF receptors (Flt-1 and Flk-1) was increased after exercise, but this increase could be inhibited by NOS inhibitor, indicating that NO may be involved in regulating VEGF expression (Gavin and Wagner, 2002). Lee et al. (Lee et al., 2000) demonstrated that NO promoted endothelial cell migration and differentiation through upregulating αvβ3 integrin expression. If αvβ3 integrin function was inhibited in endothelial cells, even in the presence of VEGF, the downstream signaling pathway of VEGFR2 was damaged, so as the migration and adhesion of endothelial cells (Danilucci et al., 2019). Ranjbar et al. (Ranjbar et al., 2016) showed that the effect of aerobic exercise on myocardial artery neogenesis in rats with myocardial infarction was also correlated with increased TGF-β expression. Aerobic exercise may increase TGF-β expression to promote angiogenesis and protect cardiovascular function. After 8 weeks of aerobic training, the myocardium blood vessel density in diabetic rats increased significantly, and the expression of TGF-β and other cytokines that regulated angiogenesis in the myocardium also increased significantly (Yazdani et al., 2020).

Thus, exercise can promote angiogenesis through systemic changes in the internal environment, including blood lipids and glucose metabolism, as well as the expression of angiogenesis-related cytokines. Besides, the effect of exercise on local metabolism and cytokine synthesis, especially in the skeletal muscle, also plays an important role in promoting angiogenesis.

## 4 Exercise-induced myokines in promoting angiogenesis

### 4.1 The mechanism underlying exercise-induced expression of myokines

Myokines are proteins or polypeptides produced by muscle tissue during contraction. The earliest discovered myokine was IL-6. Since then, CXCL family members, irisin, FSTL1, and IGF-1 have been discovered gradually (Gomarasca et al., 2020; Kwon et al., 2020). Myokines can affect the function of muscle and other tissues and organs via autocrine, paracrine, or remote secretion. Myokines maintain the shape and function of muscle cells, promote the repair of damaged muscle cells and the proliferation of skeletal muscle cells through autocrine action. The paracrine effect of myokines plays an important role in exercise-induced skeletal muscle angiogenesis. Besides, myokines can also reach the liver, heart, and other organs through long-distance secretion and participate in the processes of blood glucose and lipid control as well as angiogenesis.

Exercise-induced myokine synthesis is affected by exercise intensity, type of motion, duration, and temperature. Endurance training, resistance exercise, and intermittent high-intensity aerobic exercise can all significantly increase follistatin-like protein 1 (FSTL1) protein concentration in circulation (Kon et al., 2020; Xi et al., 2020). Strength and endurance training can both increase IGF-1 concentration in the circulation of elderly men without difference (Arazi et al., 2021). In obese adolescents, serum IGF-1 concentration is reduced, 4 weeks of moderate exercise can increase the activity but not the concentration of IGF-1 (Yang et al., 2018). Although IL-6 mRNA expression in skeletal muscle of high-fat diet rats is higher than that in the control mice, it could be reduced to a much lower level with aerobic exercise (Gopalan et al., 2021; Shirvani et al., 2021). While for diabetic patients, 6 months of resistance training had no effect on serum IL-6 concentration under the same weight loss condition (Miller et al., 2017). Besides, IL-6 mRNA expression in the muscle of one hind limb of rat at 18 °C was significantly lower than that of the opposite limb at 37 °C, while CXCL1 mRNA expression was higher at 18°C than that at 37°C (Krapf et al., 2021). When human skeletal muscle cells were heated from 18 to 37°C, IL-6 and CXCL1 mRNA expression increased significantly.

So far, how exercise-induced myokine synthesis has not been demonstrated clearly. For example, the changes in NO, protein arginine methyltransferase1 (PRMT1), and histone deacetylase 5(HDAC5) all participate in the regulation of IL-6 expression after exercise. NO inhibitor could reduce exercise-induced IL-6 mRNA expression, while after administration of nitroglycerin, which could increase NO concentration, IL-6 mRNA expression increased significantly, suggesting that NO is involved in the regulation of IL-6 mRNA expression (Steensberg et al., 2007). The effect of NO on IL-6 expression may be attributed to its effect on increasing cGMP concentration. Besides, NF-κB is a transcription factor sensitive to redox reactions. Some studies have shown that NF-κB inhibition reduces the exercise-induced transcription and synthesis of IL-6 (Pedersen and Febbraio, 2008). However, other studies in obese people showed that exercise promotes the expression of IL-6 prior to NF-κB activation (Tantiwong et al., 2010). PRMT1 is an important protease involved in post-transcriptional modification of signaling molecules. It can methylate RelA, resulting in its inability to bind to DNA and consequently affect NF-κB activation (Reintjes et al., 2016). PRMT1 mRNA and protein expression levels are increased significantly after strenuous exercise, but not after long-term exercise (van Lieshout et al., 2019). In PRMT1-deficent mice, IL-6 expression in the liver was decreased, suggesting that PRMT1 is involved in the regulation of IL-6 expression (Zhao et al., 2019). Klymenko et al. (Klymenko et al., 2020) found that HDAC5 can negatively regulate IL-6 gene expression and then affect glucose uptake in skeletal muscle cells. By electrically stimulating C2C12 cells to generate a muscle contraction model, it was found that IL-6 expression in HDAC5-deficient myocytes was higher than that in wild-type cells, suggesting that HDAC5 was also involved in the regulation of exercise-induced IL-6 expression.

### 4.2 The effect and mechanism of exercise-induced myokines in promoting angiogenesis

Exercise-induced myokines have been found to play important roles in promoting angiogenesis in the skeletal muscle and improving ischemic symptoms in the whole body (Table 2).

**TABLE 2:** The mechanisms of myokines in promoting angiogenesis.

Myokines	Glucose and lipid metabolism			Angiogenesis-related cytokines			Other myokines
	Hyperlipidemia	Hyperglycemia	Insulin Resistance	VEGF	MMP	eNOS/NO	
IL-6	↓	↓	↓	↑	–	–	–
CXCL1	–	–	–	–	–	–	IL-6↑
CXCL10	↑	–	–	–	–	–	–
Irisin	↑	–	↑	–	–	–	CXCL1↑
FSTL1	↑	↑	↓	↑	↑	↑	IL-6↑
IGF-1	–	–	–	↑	–	–	–

↑, up-regulated; ↓, down-regulated; –, not clarified or controversial.

#### 4.2.1 IL-6

IL-6 is first thought to be a proinflammatory cytokine secreted by leukocytes. IL-6 can participate in the angiogenesis of tumor tissue, thus playing an important role in tumorigenesis and development (Ancrile et al., 2007; Lin et al., 2012). The role of IL-6 in promoting angiogenesis in tumor tissues is closely related to VEGF (Ando et al., 2019; Xiao et al., 2021). Similarly, aerobic exercise can promote the expression of IL-6 and VEGF and improve the metabolic status of the whole body in obese people. Except for that, IL-6 is also closely related to metabolic syndrome and insulin resistance (Yousefabadi et al., 2021; El-Toukhy et al., 2022). IL-6 treatment can promote glucose transporter transfer to the cell membrane surface and promote glucose absorption (Carey et al., 2006). Besides, IL-6 can induce glucagon-like peptide-1 expression, thereby improving insulin secretion and blood glucose levels (Ellingsgaard et al., 2011; Ellingsgaard et al., 2020). In obese people, IL-6 also regulates glucose metabolism and insulin release by affecting gluconeogenesis in hepatocytes to affect blood glucose (Knudsen et al., 2016; Peppler et al., 2019). In addition, it has been found that muscle-derived IL-6 improves insulin resistance through activating AMPK and inhibiting the p38/MAPK signaling pathway (Tang et al., 2019). Therefore, aerobic exercise-induced IL-6 may promote angiogenesis through increasing the expression of VEGF and improving the metabolic status of whole body.

#### 4.2.2 CXCL family proteins

CXCL family proteins have been divided into two categories according to their different domains: ELR + CXCL proteins contain a Glu-Leu-Arg sequence at the N-terminal, while ELR- CXCL proteins do not. The former includes CXCL1-3 and CXCL5-8, which binding to CXCR1 and/or CXCR2 to promote angiogenesis. The latter include CXCL4, CXCL4L1, and CXCL9-14, which can bind to CXCR3, CXCR4, CXCR5, or CXCR7 to exert an anti-angiogenic effect (Lee et al., 2018). Exercise can increase CXCL1 mRNA and protein levels in muscles earlier than IL-6, suggesting that CXCL1 may promote angiogenesis by regulating the expression of IL-6 (Pedersen et al., 2011). CXCL10, also known as interferon γ-inducible protein, is secreted by monocytes and endothelial cells and can regulate chemotaxis, cell proliferation and apoptosis (Sui et al., 2004). Animal experiments show that skeletal muscle can inhibit CXCL10 expression during contraction, and p38/MAPK signaling pathway may be involved in this regulatory process; cell experiments show that CXCL10 can inhibit endothelial cell activity (Ishiuchi et al., 2018). In the high-fat diet-induced obesity mouse model, CXCL10 expression in skeletal muscle is increased, and the capillary density is decreased, while after aerobic training, CXCL10 expression is reduced, and the capillary density is increased (Ishiuchi-Sato et al., 2020). Therefore, exercise-induced increased expression of CXCL1 and decreased expression of CXCL10 in muscles may play a synergistically role in promoting angiogenesis, however, the mechanism how they regulate angiogenesis remain largely unknown.

#### 4.2.3 Irisin

Irisin is derived from fibronectin III domain protein 5, which is regulated by the transcription factor PGC1α (Bostrom et al., 2012). The function of irisin is closely related to metabolism. In patients with gestational diabetes mellitus, the blood irisin level is significantly higher compared to healthy people, and is positively correlated with fasting insulin level (Ebert et al., 2014). Prenatal exercise can induce irisin production and promote blood glucose homeostasis and improve the metabolic disorders caused by pregnancy (Szumilewicz et al., 2017). Patients with polycystic ovary syndrome also show higher levels of irisin in serum and follicular fluid than healthy people, which is positively correlated with insulin resistance (Bousmpoula et al., 2019). Besides, a positive correlation between irisin level and islet resistance also exists in obese people. Although comprehensive intervention and weight control could not change irisin level, obese people with high irisin level showed better improvement in body fat rate, blood glucose, and triglyceride than obese people with low irisin level (Fukushima et al., 2016). However, in patients with type I diabetes, the plasma irisin level is significantly lower than that in healthy people (Tentolouris et al., 2018), thus irisin is expected to be a new treatment for diabetes (Leung, 2017). This suggested that irisin might also indirectly improve the symptoms of ischemic disease and promote angiogenesis through improving metabolism.

Indeed, irisin can repair human umbilical vein endothelial cell (HUVEC) injury caused by high glucose through the Notch signaling pathway, reduce the expression of apoptosis-related proteins and increase the expression of VEGFA in HUVECs (Wang et al., 2021). In lipoprotein-injured HUVEC, irisin promotes angiogenesis through the Akt/mTOR/S6K1/Nrf2 signaling pathway (Zhang and Cao, 2019).Wu et al. (Wu et al., 2015) also confirm that irisin increases the expression of MMP2 and MMP9 through the ERK signaling pathway and promotes angiogenesis in HUVECs and zebrafish embryos. In the myocardial infarction animal model, irisin treatment also shows myocardial protection, and better angiogenesis at the edge of infarction (Liao et al., 2019). Moreover, irisin can also stimulate adipocyte secretion of CXCL1 and improve endothelial cell adhesion (Shaw et al., 2021). Together, these results suggest that irisin has strong application value in promoting angiogenesis and improving the symptoms of ischemic diseases.

#### 4.2.4 FSTL1

FSTL1, also known as TSC-36 or FRP, is a secretory glycoprotein produced by mesenchymal tissue cells (Chaly et al., 2014). It was first considered as a pro-inflammatory protein molecule induced by TGF-β and can induce IL-6 synthesis (Shibanuma et al., 1993; Miyamae et al., 2006). FSTL1 is expressed in many cells and tissues, animals lacking the *FSTL1* gene die soon after birth (Geng et al., 2011). FSTL1 plays an important role in postnatal pulmonary vessel development, deficiency of FSTL1 in mouse endothelial cells affects pulmonary vessel remodeling (Tania et al., 2017). FSTL1 also participates in regulating metabolism, and its level is closely related to insulin resistance. In obese and diabetic patients, FSTL1 circulating level is significantly higher compared to healthy people. While FSTL1 expression is decreased significantly in hyperinsulinemia even with normal blood glucose (Xu et al., 2020). FSTL1 has been found to play an important role in promoting angiogenesis in tumor metastasis and in some ischemic diseases. Muscle-derived FSTL1 can induce cardiac angiogenesis in rats with myocardial infarction by regulating the DIP2A-SMAD2/3 signaling pathway (Xi et al., 2020). FSTL1 improves vascular endothelial cell function in ischemic tissue by affecting eNOS phosphorylation, further promoting angiogenesis (Ouchi et al., 2008). FSTL1 also regulates the gene expression of MMP1, MMP3, and MMP13 (Ni et al., 2019). Deficiency of FSTL1 decreases VEGF, α-SMA and other genes related to extracellular matrix synthesis in HUVECs, while overexpression of FSTL1 increases the expression of these genes (Niu et al., 2021). These results suggest that FSTL1 is closely related to extracellular matrix synthesis and degradation. Besides, deficiency of HDAC5 in endothelial cells of patients with Scleroderma increases FSTL1 expression and improves the ability of endothelial cells to form tubules in Matrigel, suggesting that FSTL1-mediated promotion of angiogenesis is dependent on HDAC5 (Tsou et al., 2016). Therefore, FSTL1 may be critical in promoting angiogenesis in ischemic diseases by regulating blood glucose, VEGF, and cell matrix synthesis, which can all improve the symptoms and prognosis of ischemic diseases.

#### 4.2.5 IGF-1

IGF-1 consists of four subunits: A, B, C, and D. The structures of subunits A and B are similar to that of insulin, the function of subunit C is different from that of insulin, and insulin does not have subunit D. IGF-1 can promote cell proliferation and angiogenesis and resist apoptosis (Duan et al., 2010). Although IGF-1 secretion is increased during high-intensity of aerobic training, the serum concentration of its ligand remained unchanged (Nishida et al., 2010). Clinical observation shows that IGF-1 expression level is closely related to the degree of retinal angiogenesis in diabetic patients (Hellstrom et al., 2002). In the isoproterenol-induced myocardial infarction model, IGF-1 promotes angiogenesis through inducing IL-8 expression (Haleagrahara et al., 2011). IGF-1 treatment in rats with myocardial infarction can promote VEGF expression and improve the degree of myocardial ischemia (Lisa et al., 2011). Exercise and IGF-1 supplementation can reduce the degree of diabetes-induced neuropathy and increase the expression of VEGF-A and angiogenesis (Saboory et al., 2020). In the mouse model of subclinical muscle injury, IGF-1 alone could not significantly increase angiogenesis, but IGF-1 combined with exercise could increase muscle capillary density and promote muscle regeneration (Alcazar et al., 2020). These above studies show that the angiogenesis-promoting effect of IGF-1 is closely related to VEGF expression, and IGF-1 supplementation can enhance the exercise-mediated promotion of angiogenesis.

## 5 Limitation

Although myokines have been found to promote angiogenesis, current studies regarding the detailed mechanism how exercise-induced myokines promote angiogenesis and improve the symptoms of ischemic diseases remain inadequate. First, glucose and lipid metabolism, cytokines are closely related to the angiogenesis, thus myokines that could regulate these factors are thought to have angiogenesis-promoting functions. However, the direct evidence that demonstrate the relationship between myokines and angiogenesis during exercise are missing. The molecular mechanism, such as the direct target or receptors and the downstream signaling of myokines in promoting angiogenesis need to be discovered in the future. Second, both animal and clinical research data on treating ischemic diseases using exercise or myokines are insufficient probably due to lack of effective indicators of angiogenesis except for metabolic markers and related cytokines. Although some studies have used exercise or myokines in different diseases and obtain positive results, the effect of disease severity, exercise types and the level of myokines on angiogenesis need to be demonstrated much more clearly. Third, some myokines, like interleukins and chemokines, are also proinflammatory cytokines which need to be taken into account carefully because inflammation also contribute to the development of ischemic diseases. Although their expressions might also be correlated with the level of metabolic status or even angiogenesis-related cytokines, it is hard to define the main function of these myokines in the internal environment. Therefore, future studies should clarify the effect of exercise or myokines on those intracellular and extracellular factors that directly promote angiogenesis in depth.

## 6 Summary and prospects

Promoting angiogenesis is undoubtedly an effective method to improve tissue and organ perfusion, especially for ischemic diseases that cannot be treated through surgery. The benefit and safety of exercise rehabilitation for patients with ischemic diseases have been widely recognized. During exercise, the expression of myokines that promote angiogenesis increased, including IL-6, irisin, FSTL1, CXCL1, IGF-1, while the expression of CXCL10, which inhibits angiogenesis, decreased. These myokines regulate angiogenesis-related factors, such as VEGF, NOS and MMP, which play important roles in endothelial cell matrix degradation, endothelial cell migration, and lumen formation, and synergistically promote angiogenesis. In addition, the improvement on hyperglycemia and hyperlipidemia by exercise also provides a favorable internal environment for angiogenesis.

Currently, the effect of surgical treatment is often poor for ischemic diseases caused by diffuse vascular diseases. Medical treatment mainly involves the regulation of blood glucose, blood lipids, and anti-atherosclerosis, as well as thrombosis prevention. However, there is no medication that can directly promote angiogenesis. Some clinical studies have confirmed that exercise rehabilitation is a safe and effective treatment for ischemic heart disease, stroke, and peripheral vascular diseases. Due to the promising effect of exercise rehabilitation on angiogenesis and tissue perfusion, its value in treating ischemic diseases will draw more and more attention in the future, especially in ischemic diseases caused by diffuse vascular diseases. Myokines generated during exercise have also shown their potential in promoting angiogenesis *in vivo* and are expected to be a new molecular target for the treatment of ischemic diseases. Moreover, the metabolic syndrome improvement effect of myokines can make it benefit a much more broader patients with obesity and diabetes. Therefore, it is worthy to carry on more detailed studies on the function and mechanisms of exercise and myokines in promoting angiogenesis. Future study could focus on: 1) How exercise induced myokine expressions in muscle cells, the effect of contraction pathway on epigenetic changes of myokine-related genes; 2) The direct function of myokines on angiogenesis, the receptor and downstream signaling of myokines on vascular endothelial cells, as well as the effect on organ angiogenesis using *in vivo* dynamic visual evidence; 3) The clinical data about the change of myokines before and after exercise in patients with ischemic diseases, the effect of exercise on angiogenesis in ischemic organs need to be systematically collected and carefully analyzed with the exercise type, disease severity and other disease history taken into account.
